# Serological survey of immunoglobulin G from *Toxoplasma gondii* infection in dairy goats in East Java, Indonesia

**DOI:** 10.14202/vetworld.2023.1926-1932

**Published:** 2023-09-23

**Authors:** Mira Fatmawati, Lucia Tri Suwanti, Mufasirin Mufasirin, Sulinawati Fong, Sisca Valinata, Didik Tulus Subekti, Fitrine Ekawasti, Hana A. Ali Awad

**Affiliations:** 1 Sains Veteriner Doctoral Program, Faculty of Veterinary Medicine, Airlangga University, Mulyorejo, Surabaya, East Java, Indonesia; 2 Laboratory of Public Health and Epidemiology, Faculty of Veterinary Medicine, Brawijaya University, Kalisongo, Malang District, Indonesia; 3 Division of Veterinary Parasitology, Faculty of Veterinary Medicine, Airlangga University; Mulyorejo, Surabaya, East Java, Indonesia; 4 Toxoplasma Study Group, Institute of Tropical Disease, Universitas Airlangga, Surabaya, East Java, Indonesia; 5 Veterinary Disease Investigation Center, Bandar Lampung, Lampung, Indonesia; 6 The Research Organization for Health, National Research and Innovation Agency, Bogor, Indonesia; 7 Department of Parasitology, Faculty of Veterinary Medicine, Omer Al Mukhtar University, Libya

**Keywords:** dairy goats, risk factor, seroprevalence, toxoplasma modified agglutination test

## Abstract

**Background and Aim:**

*Toxoplasma gondii* infection is a significant issue of veterinary public health because it is potentially transmitted through goat milk. Therefore, the use of control measures and routine monitoring of toxoplasmosis in dairy goats is necessary. Serological analysis using antibodies can detect *T. gondii* infection. This study aimed to conduct an epidemiological study of *T. gondii* in dairy goats using antibody detection and risk factor identification.

**Materials and Methods:**

This was a cross-sectional study. We performed a serological analysis of *T. gondii* infection in dairy goats to evaluate the prevalence of toxoplasmosis. Random sampling was performed, including 132 lactating dairy goats. *Toxoplasma*-modified agglutination test was used as a serological test for immunoglobulin G with a sensitivity of 98.55%, specificity of 86.21%, and accuracy of 94.9%. A structured questionnaire was used to collect risk factor data, which were analyzed using the Chi-square and Fisher’s exact tests. The statistical package for the social sciences v. 21 was used for statistical analyses.

**Results:**

The seroprevalence of *T. gondii* in Malang and Lumajang Regency was 100% and 90.7%, respectively. A significant difference in prevalence of *T. gondii* was observed between the two districts. Livestock management practices that significantly influenced *T. gondii* seroprevalence included water sources (p < 0.05; relative risk [RR] = 1.151; 95% confidence interval [CI]: 1.044–1.269). Farmers’ characteristics that significantly influenced *T. gondii* seroprevalence included education (p < 0.05; RR = 1.125; 95% CI: 1.037–1.221), main occupation (p < 0.05; RR = 1.118; 95% CI: 1.035–1.207), and position in the organization of dairy goats farmers (p < 0.05; RR = 1.141; 95% CI: 1.022–1.274).

**Conclusion:**

In East Java, the prevalence of *T. gondii* in dairy goats is high. This study provides detailed information regarding risk factors associated with *T. gondii* seroprevalence in dairy goats in East Java, Indonesia.

## Introduction

Goat milk is a concentrated dietary source of nutrition for humans [1]. Goat milk is believed to prevent degenerative diseases [1]. Some communities consume raw goat milk [2]. Goat milk is perishable and rapidly decays if not refrigerated; therefore, it is a potential medium for the growth of microorganisms that cause foodborne diseases, including toxoplasmosis [3, 4]. Consumption of raw goat milk can lead to toxoplasmosis in humans even if it is contaminated with tachyzoites and bradyzoites. *Toxoplasma gondii* is an obligate cellular protozoan that infects mammals, including humans.

*Toxoplasma gondii* causes toxoplasmosis and plays a significant role in veterinary public health [4]. Toxoplasmosis transmission can occur through the consumption of livestock products, including undercooked meat and raw milk [5]. Toxoplasmosis caused by goat milk was first reported in the United States in 1978 [6].

In Indonesia, *T. gondii* is prevalent in goats (15%–100%), sheep (10%–82.03%), cattle (1.59%–92.6%), poultry (3.8%–30%), free-range chicken (91.64%), ducks (6.1%–20%), and pigs (2.3%–75.9%) [7–10]. Environmental, ecological, and demographic factors are risk factors for toxoplasmosis. Management of cages, feed, drinking source, and housing influence toxoplasmosis [11].

This study aimed to serologically determine whether dairy goats produce antibodies against *T. gondii* and identify management practices and farmer characteristics that influence the prevalence of toxoplasmosis.

## Materials and Methods

### Ethical approval

This study was approved by the Animal Care and Use Committee Faculty of Veterinary Medicine, Airlangga University (Approval No. 1.KE.076.06.2021).

### Study period and location

The study was conducted from July 2021 to August 2022 in the Lumajang and Malang Regency of East Java, Indonesia. This location was selected because it has the highest number of dairy goats in East Java. The serological test was conducted at the Veterinary Disease Investigation Center, Bandar Lampung, Indonesia, which has a Reference Laboratory for Toxoplasmosis.

### Sampling technique

This cross-sectional study examined exposure events to determine the relationship among seroprevalence, distribution, infection, and exposure. The observed seroprevalence was seropositive for *T. gondii* immunoglobulin G (IgG). Of the 38 districts in East Java, two have the largest population of dairy goats: Lumajang and Malang Regency. Samples located based on smaller administrative units or villages were further conveniently selected. In each selected area, dairy goats were randomly or conveniently sampled [11].

The sample size for estimating the prevalence of IgG was calculated using the following formula n = 4 p × q/(L²) [12], where n refers to the required sample size, p is the estimated prevalence, q is the result of 1–p, and L is 5% deviation rate of the sample [13]. Subsequently, the calculated sample size was 132. Random sampling was used for sampling. Seropositive results were calculated by dividing positive serological test results by the total sample. Risk factors were identified by comparing the results of seropositive tests with farm management factors and characteristics [13, 14].

### Serological testing

Serum tests were performed using the *Toxoplasma*-modified agglutination test (To-MAT Kit –Tg; antibody detection kit against *Toxoplasma gondii*, produced by Ministry of Agriculture, Republic Indonesia with Veterinary Disease Investigation Center, Lampung, Indonesia), with sensitivity of 98.55%, specificity of 86.21%, and accuracy of 94.9% [15]. The standard *Toxoplasma*-modified agglutination test was performed according to the manufacturer’s instructions. Serum samples were diluted with diluent in a ratio of 1:20 and homogenized by vortexing. To-MAT Kit –Tg suspension (25 μL) was placed in a microtiter plate, and 25 μL of sample was added to each well. Each sample and blue non-active tachyzoite suspension for immunoglobulin M and red non-active tachyzoite suspension for IgG were mixed using a pipette. The microtiter plate was incubated overnight in a refrigerator (4°C–8°C) and examined for the presence of agglutination [16].

### Risk factors

The risk factors for toxoplasmosis were identified using a structured questionnaire. The operational definition of toxoplasmosis transmission included farm management, such as cage disease, management, other farm animals, live cats, water, and food. Farmer characteristics that influence toxoplasmosis included gender, respondents’ age category, experience as a farmer, education, main business as a farmer, occupation, ownership, division of labor, participation in an organization for dairy goats, position in the organization for dairy goats, and management system.

### Statistical analysis

All statistical analyses were performed using the statistical system for the social sciences (version 21, IBM, Armonk, NY, USA). Seroprevalence was calculated by dividing the number of seropositive samples by the total number of samples. Seroprevalence was determined using the chi-square test of association. Associations between risk factors (independent variables) and variables were assessed using linear regression. Risk estimates were made with a 95% confidence interval (CI) to assess the strength of the association between risk and infection using univariate logistic regression analysis. Differences were considered statistically significant at p < 0.05.

## Results

### Seroprevalence of toxoplasmosis in dairy goats

The seroprevalence of *T. gondii* antibodies in dairy goats was 93.9% for IgG. It was significantly higher in Malang (100%) than Lumajang (90.7%) (p = 0.050). Seropositive *T. gondii* was also observed in other breeds. Higher susceptibility was observed in the Sapera breed (100%) than other breeds, such as Senduro (96.5%), Menggolo (94.7%), and Etawa crossbreeds (90.4%) (Table-1).

**Table-1:** Seroprevalence of *Toxoplasma gondii* based on regency and goat breed.

Location and Breed	n	IgG (%)		p-value

		Positive (n = 124)	Negative (n = 8)	
Regency				
Lumajang	92	90.7	9.3	0.005
Malang	40	100.0	0.0	
Goat breed				
Senduro	57	96.5	3.5	0.555
Etawa crossbreed	52	90.4	9.6	
Sapera	4	100.0	0.0	
Menggolo	19	94.7	5.3	

Not significant at 5% level (p > 0.05). IgG=Immunoglobulin G

### Management systems that influence toxoplasmosis based on IgG detection

Water source is a system management factor that significantly influences the detection of *T. gondii* IgG. The seroprevalence of *T. gondii* was related to the source of water (p < 0.05; relative risk [RR] = 1.151; 95% CI: 1.044–1.269). Farms whose water comes from surface water showed higher IgG (100%) compared with farms with municipal water source (86.9%). Other system management factors observed include cage cleanliness, presence of cats, food sources, rearing systems, and species of animals kept in one cage (Table-2).

**Table-2:** Management system that influenced toxoplasmosis based on IgG.

Management	IgG			

	Positive (n = 124)	Negative (n = 8)	RR	p-value
Cage condition			0.927 (0.888–0.977)	
Dirty	92.7	7.3		0.351
Clean	100.0	0.0		
Presence of cats			1.098 (1.029–1.171)	
Yes	100.0	0.0		0.055
No	91.1	8.9		
Water source			1.151 (1.044–1.269)	0.002*
Surface water	100.0	0.0		
Municipality water source	86.9	13.1		
Feed			0.932 (0.1020–1.116)	0.596
Forage and supplementary feed	93.2	6.8		
Fermented and supplementary feed	100.0	0.0		
Farming system			1.067 (1.020–1.116)	
Semi-intensive	100.0	0.0		1.000
Intensive	93.7	6.3		
Other species in the farm			0.927 (0.711–1.209)	
Multispecies (cow)	87.5	12.5		0.402
Only dairy goats	94.4	5.6		

Not significant at 5% level (p > 0.05). IgG=Immunoglobulin G

Clean cages (100%) had higher IgG levels than dirty cages (92.7%). Cages with roaming cats (100%) had higher IgG levels than those with no cats (91.1%). Feed sources derived from fermented feeds and supplementary feeds showed higher IgG (100%) than those derived from grasses (93.2%). The semi-intensive livestock system had higher IgG (100%) than the intensive system (93.7%). Farms consisting of only one species, in this case, dairy goats, had higher IgG (94.4%) than multispecies farms, for example, those with dairy goats and dairy cows in one cage (87.5%).

### Farmer characteristics that influence toxoplasmosis based on IgG detection

The characteristics of farmers that influence seropositive toxoplasmosis are listed in Table-3. The prevention and control of animal diseases between farms frequently depend on good farming practices. However, farmers regularly do not implement disease control programs. This practice indicates a comprehensive description of the farmer characteristics that influence toxoplasmosis According to univariate analysis, the seroprevalence of *T. gondii* was related to farmer education (p < 0.05; RR = 1.125; 95% CI: 1.037–1.221), main occupation (p < 0.05; RR = 1.118; 95% CI: 1.035–1.207), and position in the organization of dairy goats (p < 0.05; RR = 1.141; 95% CI: 1.022–1.274). Farmers with low education levels (100%) had higher IgG levels than those who graduated from high school (88.9%). Farmers whose main occupation was not goat farming had lower IgG levels than farmers whose main occupation was goat farming (89.5%). Farms managed by women (100%) had higher IgG levels than those managed by men (93.4%). Immunoglobulin G detection in adult farmers (96.9%) was higher than that in senior farmers (93.0%). Beginner breeders (100%) had higher IgG levels than experienced farmers (89.5%). Regarding livestock ownership, farms managed by farm laborers (100%) had higher IgG levels than those managed by the owners (93.0%). Farmer participation in dairy goat breeder organizations affected the results of *T. gondii* IgG detection. Farmers who did not participate in organizations had higher IgG detection test results (100%) than those who participated in organizations (93.3%). Farms where the owner’s position in the organization was only as a member had higher IgG detection test results (98.8%) than those whose farmers were leaders in the organization (86.6%).

**Table-3:** Characteristic of farmer that influenced toxoplasmosis.

Characteristic	IgG			

	Positive (n = 124)	Negative (n = 8)	RR	p-value
Gender				
Women	100.0	0.0	1.071	1.000
Man	93.4	6.6	(1.021–1.123)	
Working age category				
Adult (17–55)	96.9	3.1	1.042	0.679
Senior (up to 55)	93.0	7.0	(0.959–1.131)	
Education				
Middle (No - graduated high school)	100.0	0.0	1.125	0.008*
Secondary (Graduated high school)	88.9	11.1	(1.037–1.221)	
Main occupation				0.021*
Others	100.0	0.0	1.118	
Goats farmer	89.5	10.5	(1.035–1.207)	
Experience as a farmer				
Beginner	100.0	0.0	1.081	0.352
Advance	92.5	7.5	(1.024–1.141)	
Ownership				
Worker	100.0	0.0	1.075	0.596
Owner	93.0	7.0	(1.022–1.130)	
Participation in organization of dairy goats				
No	100.0	0.0	1.071	1.000
Yes	93.3	6.7	(1.021–1.125)	
Position in organization for dairy goats				
Member	98.8	1.2	1.141	0.006*
Chairman	86.5	13.5	(1.022–1.274)	

Not significant at 5% level (p > 0.05). IgG=Immunoglobulin G, RR=Risk ratio

* RR value is considered significant

## Discussion

### Seroprevalence of toxoplasmosis in dairy goats

This study showed that Malang Regency had higher toxoplasmosis prevalence than Lumajang Regency, indicating that seroprevalence was influenced by location. Other studies have reported that the seroprevalence of toxoplasmosis is correlated with location [17–19].

This study is related to the environmental disease that spreads depending on rainfall and temperature [11, 20, 21]. Moisture and heat are important factors for *T. gondii* sporulation and survival. Precipitation varies geographically. High rainfall occurs in coastal areas from South Java to North Java. It has an impact on the environmental temperature that is becoming increasingly humid. Differences in rainfall and temperature can cause significant differences in prevalence between the two districts. Rainfall and temperatures are higher in Malang Regency (Ampelgading subregency) than Lumajang Regency (Senduro subregency), according to data from the Meteorological, Climatological, and Geophysical Agency, Indonesia.

In this study, the Sapera breed had a higher prevalence of antibodies against *T. gondii* than other breeds. Consistently, Tonouhewa *et al*. [19] reported that breed did not influence the frequency of infection. In contrast, Zhao *et al*. [22] reported a significant racial difference in susceptibility to toxoplasmosis. This difference may be due to differences in species resistance to parasitic infections.

Differences in serological test results are related to the method used. Different types of serological testing for toxoplasmosis allow for different test results. In this study, the *Toxoplasma-*modified agglutination test was used. *Toxoplasma*-modified agglutination test was developed by the Ministry of Agriculture, Republic of Indonesia for toxoplasmosis testing [16]. *Toxoplasma*-modified agglutination test has 98.55% sensitivity, 86.21% specificity, and 94.9% accuracy. The To-MAT kit Tg has 74.1% compatibility with the Toxotest (IDEXX) ELISA kit and 76% compatibility with immunoblotting [15]. According to Sadek *et al*. [23], antibodies against *Toxoplasma* can be detected 2 weeks after the onset of infection and can persist in the host’s body at low levels throughout life. The seroprevalence of toxoplasmosis in goats is influenced by immunity, time of infection, and genetics. *T. gondii* is infective when immunity is low [24]. During low immunity, *T. gondii* is activated and spreads through the blood vessels to reach the mammary glands and is secreted through milk [12, 25].

### Livestock management practices that influence *T. gondii* based on IgG detection

The presence of cats around cages increases the possibility of infection from definitive hosts to intermediate hosts. *Toxoplasma gondii* could be transmitted through cat feces-contaminated water. Water from wells is also a source of *T. gondii* transmission. Leaving water in watering troughs for several days and filling them up as they become empty makes them potentially accessible to cats [26]. A study reported that surface water, as a source of drinking water for dairy goats, is a risk factor for *T. gondii* infection [5, 26–28]. Cats are the definitive host of *T. gondii*. They excrete feces with oocytes on their surface, which can be a source of infection. Therefore, an association between the presence of cats and seropositive toxoplasmosis exists [29]. The same study reported that the presence of cats and domestic animals and access to drinking sources for livestock are risk factors for toxoplasmosis [4, 11]. The serological and risk factors affecting toxoplasmosis in goats and sheep are presented in Table-4 [4, 17–20, 28–33].

**Table-4:** Prevalence and risk factors for toxoplasmosis in sheep and goats.

References	Methods	Prevalence of *Toxoplasma gondii*		Significant factor that influencing toxoplasmosis

		Sheep (%)	Goat (%)	
Chiang *et al*. [4]	ELISA		32.2	The improvement of flooring material or through cleaning periodic disinfection and maintenance or dryness on the floor are highly recommended for the prevention
Mbari *et al*. [17]	Latex particle agglutination test	8.55	18.24	Toxoplasma prevalence according to region department
Othman and Zuheir [18]	Indirect ELISA	13.4		The seroprevalence was influenced by the location of the goat herds
Tonouhewa *et al*. [19]	ELISA	1.4	53.0	Age, gender
Ahmed *et al*. [20]	ELISA	26.2	42.8	The seroprevalence was higher in women than men; a wide variation in the seroprevalence of *T. gondii*
Rêgo *et al*. [28]	ELISA	48.7	40.5	Cat feeding, the number of domestic dogs on the farm, gender
Sechi *et al*. [29]	IFAT	33.97		Herd size, water source, and access of cats to water administered to animals
Hotea *et al*. [30]	ELISA	50.64	75.0	Biological characteristic (age, sex, and breed) rearing management (cats were present and water)
Udonsom *et al*. [31]	ELISA		28.5	The presence of cat
Magalhães *et al*. [32]	Indirect immunofluorescence	85.0	10.7	Contact with sheep, extensive farming system, water source, more than three cats per farm, presence of rat in the feed storage location
Alvarado-Esquivel *et al*. [33]	Modified agglutination test		15.2	Seroprevalence varied according to age, municipality, altitude, and climate but not breed

ELISA=Enzyme-linked immunosorbent assay, IFAT=Indirect fluorescent antibody test

### Characteristics of respondents who influence *T. gondii* based on IgG detection

Farmers have unique demographic features. Socio-psychological factors, age, gender, education, personality, and previous experiences of farmers influence disease prevalence [34]. These individual characteristics contribute to farmers’ perceptions of animal health, prevention and control strategies, and influence decision-making. Understanding the farmer’s mindset and factors that influence mindset is critical for motivating them to change. However, interventions to change farmer behavior must acknowledge that farmers are not homogeneous and cannot be convinced by relying only on educational arguments [30]. Furthermore, farmers’ context (e.g., laws and regulations, market prices, or quality programs) can affect decision-making by inhibiting or facilitating recommended management changes. Therefore, providing a “one-size-fits-all” solution is impossible. The education and engagement of personnel can be a major challenge due to their different languages and cultural backgrounds. It could increase compliance with recommended procedures for hand hygiene and biosecurity strategies. Farm employees have reasons for performing recommended biosecurity measures. However, they need more knowledge, better attitudes, and more compliance on the farm. Farm employees must improve their adherence to farmer’s management [30, 34].

## Conclusion

The results show that in East Java, high antibody detection against *T. gondii* was observed in dairy goats with a seroprevalence of 95.4%. Livestock management practices that significantly influenced *T. gondii* seroprevalence included water source. Farmers’ characteristics that significantly influenced *T. gondii* seroprevalence included education, main occupation, and position in the organization of dairy goats.

## Authors’ Contributions

MF, LTS, MM, SF, SV, DTS, FE, and HAAA: Study conception and design. MF and LTS: Contributed to writing, revision, and editing of the manuscript. MM: Critically reviewed and revised the manuscript. SF and SV: Conducted the experiments and analyzed the data. DTS and FE: Reviewed and edited the manuscript. HAAA: Acquisition of data and reviewed and edited the manuscript. All authors have read, reviewed, and approved the manuscript.

## References

[1] Hammam A.R.A, Salman S.M, Elfaruk M.S, Alsaleem K.A (2021). Goat Milk:Compositional, Technological, Nutritional, and Therapeutic Aspects (preprint).

[2] Icoutchika K.L, Ahozonlin M, Mitchikpe E, Bouraima O, Aboh A, Dossa L (2022). Socio-economic determinants of goat milk consumption by rural households in the Niger valley of Benin and implications for the development of a smallholder dairy goat program. Front. Sustain. Food Syst.

[3] Boughattas S (2015). Toxoplasma infection and milk consumption:Meta-analysis of assumptions and evidence. Crit. Rev. Food Sci. Nutr.

[4] Chiang S.H, Huang H.H, Chou C.C, Chu C.S, Shih W.L, Lai J.M, Lin H.C, Yang W.C, Lee H.H, Tsai Y.L, Su Y.C (2020). Epidemiological survey of *Toxoplasma gondii* and *Neospora caninum* infections in dairy goats in Central-Southern Taiwan. J. Vet. Med. Sci.

[5] Stelzer S, Basso W, Benavides S.J, Ortega-Mora L.M, Maksimov P, Gethmann J, Conraths F.J, Schares G (2019). *Toxoplasma gondii* infection and toxoplasmosis in farm animals:Risk factors and economic impact. Food Waterborne Parasitol.

[6] Skinner L.J, Timperley A.C, Wightman D, Chatterton J.M, Ho-Yen D.O (1990). Simultaneous diagnosis of toxoplasmosis in goats and goatowner's family. Scand J Infect Dis.

[7] Iskandar T (2008). Toxoplasmosis disease in goats and sheep in Java [Penyakit Toksoplasmosis pada Kambing dan Domba di Jawa]. Wartazoa.

[8] Fatmawati M, Suwanti L.T, Mufasirin M (2022). Review:Potential transmission of toxoplasmosis through consumption of raw goat's milk. Adv. Anim. Vet. Sci.

[9] Fatmawati M, Padaga M.C, Mayashinta D.K, Aini F.N (2021). Risk factors for toxoplasmosis in goat and sheep that influence in human infection. J. Ilmu-Ilmu Peternakan.

[10] Wulandari R, Suwandi J.F, Mutiara H, Hanriko R (2019). Seroprevalence of *Toxoplasma gondii* in cattle in Bandar Lampung City [Seroprevalensi *Toxoplasma gondi*i pada hewan ternak sapi di kota bandar Lampung]. J. Agromed.

[11] Retmanasari A, Widartono B.S, Wijayanti M.A, Artama W.T (2017). Prevalence and risk factors for toxoplasmosis in middle Java, Indonesia. EcoHealth.

[12] Bezerra M.J.G, Kim P.C.P, Moraes É.P.B.X, Sá S.G, Albuquerque P.P.F, Silva J.G, Alves B.H.L.S, Mota R.A (2015). Detection of *Toxoplasma gondii* in the milk of naturally infected goats in the Northeast of Brazil. Transbound. Emerg. Dis.

[13] Sumiarto B, Budiman S (2018). Epidemiologi Veteriner Analitik.

[14] Thrusfield M (2015). Veterinary Epidemiology.

[15] Valinata S, Sulinawati F, Subekti D.T (2020). Evaluation of test performance and suitability between the modified toxoplasma agglutination test and various commercial serological test kits [Evaluasi performa dan kesesuaian uji antara uji aglutinasi toxoplasma modified agglutination test dengan berbagai kit uji serologis komersial]. J. Vet.

[16] Ministry of Agriculture (MoA) Republic Indonesia and Veterinary Disease Investigation Center Lampung T*o*-MAT Kit-Tg Toxoplasma Modified Agglutination Test, Antibody Detection against *Toxoplasma gondii*.

[17] Mbari K.B, Biego G.G, Edouardk N, Oliver G.O, Guy-gerard K.K (2018). Sheep and goats toxoplasmosis prevalence in northern borders areas of cote divoire:Case of bagoue and tchologo regions. Int. J. Adv. Res.

[18] Othman R.A, Zuheir I.A (2014). Seroprevalence of *Toxoplasma gondii* in goats in two districts in Northern palestine 5. 2012. Walailak J Sci Tech.

[19] Tonouhewa A.B.N, Akpo Y, Sherasiya A, Sessou P, Adinci J.M, Aplogan G.L, Youssao I, Assogba M.N, Farougou S (2019). A serological survey of *Toxoplasma gondii* infection in sheep and goat from Benin, West-Africa. J. Parasit. Dis.

[20] Ahmed H, Malik A, Arshad M, Mustafa I, Khan M.R, Afzal M.S, Ali S, Mobeen M, Simsek S (2016). Seroprevalence and spatial distribution of toxoplasmosis in sheep and goats in North-Eastern region of Pakistan. Korean J. Parasitol.

[21] Shapiro K, Bahia-Oliveira L, Dixon B, Dumètre A, de Wit L.A, VanWormer E, Villena I (2019). Environmental transmission of *Toxoplasma gondii*:Oocysts in water, soil and food. Food Waterborne Parasitol.

[22] Zhao G.H, Zhang M.T, Lei L.H, Shang C.C, Cao D.Y, Tian T.T, Li J, Xu J.Y, Yao Y, Chen D.K, Zhu X.Q (2011). Seroprevalence of *Toxoplasma gondii* infection in dairy goats in Shaanxi Province, Northwestern China. Parasit Vectors.

[23] Sadek O.A, Abdel-Hameed Z.M, Kuraa H.M (2015). Molecular detection of *Toxoplasma gondii* DNA in raw goat and sheep milk with discussion of its public health importance in assiut governorate. Assiut Vet. Med. J.

[24] Abdelrahman M, El-Manyawe S.A.M.K, Saba S (2012). Occurrence of toxoplasma antibodies in caprine milk and serum in Egypt. Assiut Vet., Med. J.

[25] Camossi L.G, Greca-Júnior H, Corrêa A.P, Richini-Pereira V.B, Silva R.C, DaSilva A.V, Langoni H (2011). Detection of *Toxoplasma gondii* DNA in the milk of naturally infected ewes. Vet. Parasitol.

[26] Vesco G, Buffolano W, Chiusa S, Mancuso G, Caracappa S, Chianca A, Villari S, Currò V, Liga F, Petersen E (2007). *Toxoplasma gondii* infections in sheep in sicily, Southern Italy. Vet. Parasitol.

[27] Martínez-Rodriguez L.C, Tafur-Gómez G.A, Guzman-Barragan B.L (2020). *Toxoplasma gondii* in small ruminants in Northeastern areas of Colombia:seroprevalence and risk factors. Parasite Epidemiol. Control.

[28] Rêgo W.M.F, Paula N.R.O, Vitor R.W.A, Silva R.A.B, Diniz B.L.M, Sousa M.M, Coelho W.A.C, Porfirio K.P, Pinheiro R.R, Alves F.S.F, Cavalcante A.C.R, Cardoso J.F.S (2016). Risk factors for *Toxoplasma gondii* infection in goats and sheep raised in the state of Piauíin northeast Brazil. Small Rumin. Res.

[29] Sechi P, Ciampelli A, Cambiotti V, Veronesi F, Cenci-Goga B.T (2013). Seroepidemiological study of toxoplasmosis in sheep in rural areas of the Grosseto district, Tuscany, Italy. Ital. J. Anim. Sci.

[30] Hotea I, Herman V, Tîrziu E, Colibar O, Brudiu I, Sîrbu C, Dărăbuş G (2021). Seroprevalence and risk factors for *Toxoplasma gondii* infection in sheep and goats from Romania. Parasitologia.

[31] Udonsom R, Supanta J, Tanglakmankhong O, Ngoenphisutsin K, Nishikawa Y, Fereig R.M, Jirapattharasate C (2021). *Toxoplasma gondii* and *Neospora caninum* prevalence and risk factors on goat farms in Kanchanaburi province. Thailand. Vet. Integr. Sci.

[32] Magalhães F.J.R, Ribeiro-Andrade M, de Alcântara A.M, Pinheiro Júnior J.W, de Sena M.J, Porto W.J.N, da Vieira R.F.C, Mota R.A (2016). Risk factors for *Toxoplasma gondii* infection in sheep and cattle from fernando de Noronha Island, Brazil. Rev. Bras. Parasitol. Vet.

[33] Alvarado-Esquivel C, Silva-Aguilar D, Villena I, Dubey J.P (2013). Seroprevalence of *Toxoplasma gondii* infection in dairy goats in Michoacán State, Mexico. J. Parasitol.

[34] Ritter C, Jansen J, Roche S, Kelton D.F, Adams C.L, Orsel K, Erskine R.J, Benedictus G, Lam T.J.G.M, Barkema H.W (2017). Invited review:Determinants of farmers'Adoption of management-based strategies for infectious disease prevention and control. J. Dairy Sci.

